# Quantitative real-time RT-PCR validation of differential mRNA expression of *SPARC*, *FADD*, *Fascin*, *COL7A1*, *CK4*, *TGM3*, *ECM1*, *PPL *and *EVPL *in esophageal squamous cell carcinoma

**DOI:** 10.1186/1471-2407-6-33

**Published:** 2006-02-02

**Authors:** Nan Hu, Luxia Qian, Ying Hu, Jian-Zhong Shou, Chaoyu Wang, Carol Giffen, Quan-Hong Wang, Yuan Wang, Alisa M Goldstein, Michael Emmert-Buck, Philip R Taylor

**Affiliations:** 1 Division of Cancer Epidemiology and Genetics, National Cancer Institute, Bethesda, MD 20892, USA; 2 Center for Cancer Research, National Cancer Institute, Bethesda, MD, 20892, USA; 3 Cancer Institute & Hospital, Chinese Academy of Medical Sciences, Beijing 100021, PR China; 4 Information Management Services, Inc, Silver Spring, MD 20904, USA; 5 Shanxi Cancer Hospital, Taiyuan, Shanxi 030013, PR China

## Abstract

**Background:**

Esophageal squamous cell carcinoma (ESCC) is one of the most malignant tumors and typically presents at an advanced and rapidly fatal stage. To better understand the role of genetics in the etiology and prevention of ESCC and to identify potential susceptibility genes as well as early detection markers, we previously compared tumor and matched normal tissues from ESCC patients from a high-risk area of China using cDNA expression microarrays and identified 41 differentially-expressed genes (13 over-expressed and 28 under-expressed).

**Methods:**

In the current study, we validated and quantitated differential mRNA expression in a sample of nine of these 41 genes, including four that were over-expressed (*SPARC*, *FADD*, *Fascin*, *COL7A1*), and five that were under-expressed (*CK4*, *TGM3*, *ECM1*, *PPL*, *EVPL*), in 75 new ESCC patients using quantitative Real-time RT-PCR and the 2^-ΔΔCT ^method to examine both tumor and matched normal tissue. In addition, we examined expression patterns for these genes by selected demographic and clinical characteristics.

**Results:**

Four previously over-expressed (tumor ≥2-fold normal) genes were all increased in the majority of new ESCC patients: *SPARC *was increased in 71% of patients, *Fascin *in 70%, *FADD *in 63%, and *COL7A1 *in 57%. Five previously under-expressed (tumor ≤0.5-fold normal) genes similarly showed decreased mRNA expression in two-thirds or more of patients: *CK4 *was decreased in 83% of patients, *TGM3 *in 77%, *ECM1 *in 73%, and *PPL *and *EVPL *in 67% each. In subset analyses, associations with age (for *COL7A1*), family history (for *PPL *and *ECM1*), and alcohol use (for *SPARC *and *Fascin*) were also noted.

**Conclusion:**

These data indicate that these nine genes have consistent differential mRNA expression, validating results of our previous cDNA array results, and affirming their potential role in the early detection of ESCC.

## Background

Esophageal squamous cell carcinoma (ESCC) is one of the most malignant tumors and typically presents at an advanced and rapidly fatal stage. To better understand the role of genetics in the etiology and prevention of ESCC and to identify potential susceptibility genes as well as early detection markers, we previously compared tumor and matched normal tissues from ESCC patients from a high-risk area of China using cDNA expression microarrays and identified 41 differentially-expressed genes (13 over-expressed and 28 under-expressed) [1].

Among these 41 differentially-expressed genes are *SPARC *(secreted protein acidic and rich in cysteine), *COL7A1 *(collagen type VII, α), and *ECM1 *(extracellular matrix protein), all of which are involved in extracellular matrix functions [2-4]. Other of these differentially-expressed genes (eg, *Fascin *and cytokeratin 4, also called *KRT4 *or *CK4*) are involved in the formation of actin filaments and cytoskeleton structure [5,6].*PPL *(periplakin) and *EVPL *(enveloplakin) are both members of the *plakin *family [7-9]. *PPL *is expressed in stratified squamous epithelia while *EVPL*, a candidate gene for the tylosis esophageal cancer syndrome, is exclusively expressed in stratified squamous epithelia. Both *PPL *and *EVPL *have desmosome components and, in conjunction with *TGM3 *(transglutaminase) and *cystatin A*, they help to maintain an intact cell surface interface [10,11]. *FADD *(Fas-associated death domain) interacts with *FasL *and *Caspase-8 *to initiate the Fas signaling complex which leads to apoptosis [12]. All nine of these genes identified in our previous study [1] are involved in important cellular processes, and their altered expression in ESCC suggests that they are candidate molecular markers that may have a role in prevention and early detection strategies in ESCC.

Array technologies are comprehensive and relatively accurate ways to simultaneously analyze the expression of thousands of genes, and these technologies have been used to clarify gene expression changes in many human malignancies. However, the results from microarrays are potentially influenced by many external factors, including array production itself, RNA extraction methods, the probes used for labeling, hybridization conditions, image analysis, etc. Further, most studies of this type are based on relatively small sample sizes, including our study [1]. Thus, genes identified as differentially expressed in such initial discovery efforts need to be confirmed using alternative methods and larger sample sizes before they can be considered validated and advanced for testing as early detection markers. This confirmation is a key initial step in the validation process for selecting genes for future study as potential markers of susceptibility or early disease.

The main goal of the current study was to validate the differential mRNA expression of nine selected genes (*SPARC*, *FADD*, *Fascin*, *COL7A1*, *CK4*, *TGM3*, *ECM1*, *PPL *and *EVPL*) in a relatively large sample of ESCC cases using quantitative Real-time RT-PCR. A secondary goal was to determine if expression patterns for these nine genes varied by selected demographic and clinical characteristics.

## Methods

### Patient selection and sample collection

This study was approved by the Institutional Review Boards of the Shanxi Cancer Hospital and the U.S National Cancer Institute (NCI). Patients presenting from 1996 to 2001 to the Shanxi Cancer Hospital in Taiyuan, Shanxi Province, People's Republic of China, who were diagnosed with ESCC and considered candidates for curative surgical resection were identified and recruited to participate in the study. None of the patients had prior therapy and Shanxi was the ancestral home for all. After obtaining informed consent, patients were interviewed to obtain information on demographic and lifestyle cancer risk factors (eg, smoking, alcohol drinking, family history (FH) of upper gastrointestinal (UGI) cancer) and clinical data. Tumor tissue obtained during surgery was snap-frozen in liquid nitrogen, along with matched normal tissue, and stored at -130 C until used. The 75 patients evaluated here were selected based on the quantity and quality of their total RNA from among 110 who had sufficient tumor and matched normal tissues.

### Real-time quantitative RT-PCR

Total RNA was extracted from frozen tumor and matched normal tissues using TRIzol reagent (Invitrogen, CA, USA) in accordance with the manufacturer's instructions. RNA quality and quantity were determined using the RNA 6000 Labchip/Agilent 2100 Bioanalyzer (Agilent Technologies, Germantown, MD). RNA purification was performed according to the manufacturer's instructions for the RNeasy Mini Kit (Qiagen Inc., Valencia, CA) and Rnase-Free Dnase Set digestion (Qiagen Inc., Valencia, CA). Reverse transcription of RNA was performed by adding 5 μg total RNA, 1 μl of Oligo (dT)_12–18 _(500 μg/ml), 1 μl (200 units) of Superscript II reverse transcriptase, 1 μl (2 units) of *E. coli *Rnase, and 1 μl 10 mM dNTP (Invitrogen, Carlsbad, CA).

All real-time PCR reactions were performed using an ABI Prism 7000 Sequence Detection System (Perkin-Elmer Applied Biosystems, Foster City, CA). All primers and probes of nine target genes and an internal control gene glyceraldehyde-3-phosphate dehydrogenase (*GAPDH*) were designed by Perkin-Elmer Applied Biosystems Perkin-Elmer Applied Biosystems, Foster City, CA) (Table 1). A singleplex reaction mix was prepared according to the manufacture's protocol, as described previously [13]. The thermal cycling conditions included an initial denaturation step at 95°C for 10 min, 40 cycles at 95°C for 15 s, and 60°C for one min.

Using these quantitative methods requires that the PCR efficiencies of all genes be similar and, preferably, ≥90%. Efficiency was measured using a standard curve generated by serial dilutions of the RNA. Consequently, the initial RNA concentration of 100 ng/μl was serially diluted 10-fold (100 ng, 10 ng, 1 ng, 0.1 ng, and 0.01 ng) for the real-time PCR assay according to the standard protocol of Applied Biosystems. The relative standard curve quantitation method was previously described [14]. The PCR efficiency (E) was calculated by the formula: E = 10 ^(1/-slope) ^- 1, and ranged from 90–100% in the different assays (a slope of -3.32 is equivalent to 100% PCR efficiency) [14,15].

### Analysis of gene expression using the 2^-ΔΔCT ^method

Details of the 2^-ΔΔCT ^method have been previously described [14,16]. Briefly, the mean target gene mRNA expression level for the three mRNA measurements was calculated. The 2^-ΔΔCT ^method was used to calculate relative changes in gene expression determined from real-time quantitative PCR experiments. In the present study, the data are presented as the fold change in target gene expression in tumors normalized to the internal control gene (*GAPDH*) and relative to the normal tissue control (matched normal as calibrator). Results of the real-time PCR data were represented as C_T _values, where C_T _was defined as the threshold cycle number of PCRs at which amplified product was first detected. There is an inverse correlation between C_T _and amount of target: lower amounts of target correspond to a higher C_T _value, and higher amounts of target have lower C_T _values. The average C_T _was calculated for both the target genes and *GAPDH *and the ΔC_T _was determined as (the mean of the triplicate C_T _values for the target gene) minus (the mean of the triplicate C_T _values for *GAPDH*). The ΔΔC_T _represented the difference between the paired tissue samples, as calculated by the formula ΔΔC_T _= (ΔC_T _of tumor - ΔC_T _of normal). The N-fold differential expression in the target gene of a tumor sample compared to the normal sample counterpart was expressed as 2^-ΔΔCT ^[14,16]. In the present study, increased mRNA expression was defined as N-fold ≥2.0, "normal" expression was an N-fold ranging from 0.5001 to 1.9999, and decreased mRNA expression was N-fold ≤0.5.

### Data analysis

Hierarchical cluster analysis and visualization were generated by Cluster and TreeView software [17].

The associations between mRNA expression level of target genes and life style risk factors as well as clinical/pathological characteristics listed in Table 2 were evaluated by using chi-square tests for analysis of categorical variables and Wilcoxon rank sum tests for analysis of continuous variables (Statistical Analysis Systems (SAS), SAS Corp., NC). All *P*-values were two-sided and considered statistically significant if *P *< 0.05.

## Results

A total of 75 pairs of tumor and matched normal tissues from ESCC patients were examined for mRNA expression of nine genes using quantitative Real-time RT-PCR. Table 2 shows the characteristics of patients in the present study.

**Figure 1:**
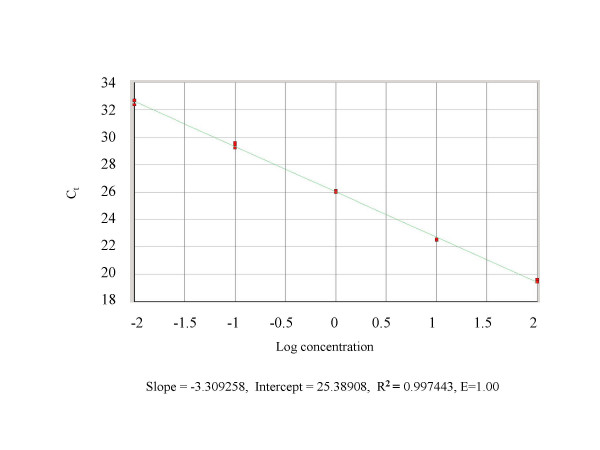
The relative standard curve using the *TGM3 *gene, primers, and probes amplified with 100, 10, 1, 0.1 and 0.01 ng of total RNA. Each sample was run in triplicate (red square). The average C_T _values (y-axis) are plotted against the logarithm of the input amount of RNA (x-axis) added to each sample. PCR efficiency (E) = (10^-1/slop ^- 1). It shows a linear relationship between RNA concentration and the C_T _value of reverse transcription real-time PCR reaction for *TGM3*.

**Figure 2:**
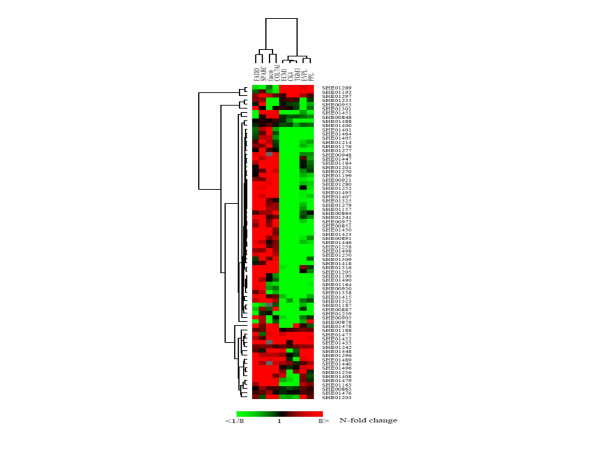
The mRNA expression of nine genes in 75 ESCC patients using hierarchical cluster with the average linkage uncentered correlation method. The cases with increased mRNA expression are shown in red; cases with decreased mRNA expression are in shown in green.

**Figure 3:**
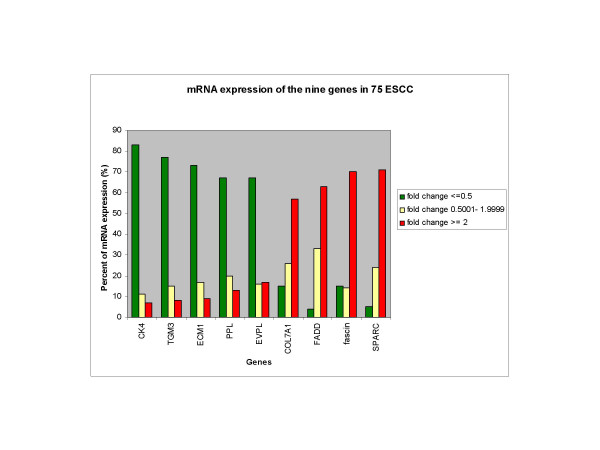
The distribution of mRNA expression of nine genes in 75 ESCC patients. Increased expression (≥2 N-fold) is shown in red; "normal" expression (N-fold from 0.50001 to 1.9999) is shown in yellow; decreased expression (N-fold ≤0.5) is shown in green.

The PCR efficiency (E) for the nine genes was measured using standard curves generated by serial dilutions of RNA and ranged from 90% to 100% for the nine genes tested (Table 1 and Fig 1). Quantitative RT-PCR analyses of mRNA levels were performed using paired normal-tumor mRNA extracts and the 2^ΔΔCT ^method. The mRNA expression values for all 75 ESCC patients tested are shown in Fig 2 and Fig 3 and summarized in Table 3. Briefly, all four genes over-expressed in our initial study also had increased mRNA expression (≥2-fold in tumor versus normal) in the majority of the new ESCC patients tested (shown in red in Fig 2): *SPARC *was increased in 71% of cases, *Fascin *in 70%, *FADD *in 63%, and *COL7A1 *in 57%. Likewise, the five genes previously identified in our initial cDNA study as under-expressed (≤0.5-fold in tumor versus normal) also had decreased mRNA expression in two-thirds or more of the new patients examined (shown in green in Fig 2): *CK4 *was decreased in 83% of cases, *TGM3 *in 77%, *ECM1 *in 73%, and *PPL *and *EVPL *decreased in 67% each. Overall, the results of the current study indicate a reasonably consistent pattern for mRNA expression in these nine genes in ESCC patients (Table 3), and are quite compatible with the findings of our earlier cDNA microarray study [1].

The range of mRNA expression found using the quantitative real-time RT-PCR method was very broad (Table 3). Among the nine genes, *TGM3 *had the greatest range of mRNA expression (from 0.000 to 35857-fold difference), while *PPL *had the smallest range (0.0076 to 29.9953-fold difference). Several ESCC cases had unusually different mRNA expression patterns for a few genes. These outliers had not only opposite expression pattern, but also had extremely low (<0.01-fold) or high mRNA expression level (>100-fold change). For example, 70% of cases had increased mRNA expression for *Fascin*, but SHE1233 and SHE1338 had very low *Fascin *mRNA expression (0.0033 and 0.0091-fold change, respectively). In addition, most cases had decreased *ECM1*, *TGM3 *and *EVPL *mRNA expression, but SHE1992 had increased mRNA expression for *ECM1 *and *TGM3 *(139- and 1050-fold changes, respectively), SHE1289 had extremely high mRNA expression for *TGM3 *(35857-fold) and *CK4 *(467-fold change), and SHE1432 had increased mRNA expression for *EVPL *(136-fold change) and *TGM3 *(99-fold change). This phenomenon regarding extreme variation in mRNA expression strongly suggests that there is heterogeneity in ESCC, even when all the patients studied come from a geographically similar high-risk population [18].

We also explored the relationships between mRNA expression levels for each target gene and age, gender, smoking, alcohol drinking, FH of UGI cancer, and the four clinical characteristics listed in Table 2 and identified five potential associations using the nominal *P*-value cutoff of 0.05. Family history of UGI cancer has been of particular interest to us since we previously showed that FH (+) cases had greater allelic loss than FH (-) cases [19,20] and that gene expression profiles varied by FH status [1]. As a result, we examined expression of all nine genes by FH status in this study (Table 4). Two genes, *PPL *and *ECM1*, had lower mRNA expression in FH (+) ESCC cases than in FH (-) cases (78% versus 62% under-expression, respectively, for *PPL*, Wilcoxon rank sum test *P *= 0.040; and 87% versus 67% under-expression, respectively, for *ECM1*, Wilcoxon rank sum test *P *= 0.038). Expression of two other genes, *TGM3 *and *EVPL*, showed lower median values in FH (+) versus FH (-) cases, although the results did not achieve statistical significance (0.05 <*P *< 0.10 for both). Interestingly, the median values for all nine genes were universally more extreme in FH (+) than FH (-) cases (ie, the five under-expressed were more under-expressed in FH (+) cases; the four over-expressed were more over-expressed in FH (+) cases). Older age was associated with over-expression of *COL7A1 *(63% over-expression in cases 56+ years of age versus 50% over-expression in cases <56 years old, Wilcoxon rank sum test *P *= 0.030). Alcohol use was associated with reduced over-expression of both *SPARC *and *Fascin *(over-expression of 40% in alcohol users versus 75% in non-users for *SPARC*, χ^2^_2 df _= 8.38, *P *= 0.015; over-expression of 50% in alcohol users versus 74% in non-users for *Fascin*, χ^2^_2 df _= 13.15, *P *= 0.001). No other significant associations were found for these variables.

## Discussion

Our interest in further validation of initial results from DNA microarray technology and quantitation of measurements to better understand the nature of differences led us to perform quantitative Real-time RT-PCR analyses of nine of the differentially expressed genes we identified in a previous study [1]. Reassuringly, results from the current study show that the differential expression patterns for these nine genes are similar to those seen with the 8 k cDNA array [1]. Four genes that were over-expressed in our first study (*SPARC*, *FADD*, *Fascin*, and *COL7A1*) also showed increased mRNA expression in the majority of the new ESCC patients tested here, while five genes previously under-expressed (*CK4*, *TGM3*, *ECM1*, *PPL*, and *EVPL*) were also under-expressed in two-thirds or more of new cases. Taken together, the results from these two studies indicate that the differential expression observed is highly consistent.

The mRNA expressions of the nine genes examined had wide ranges, suggesting that quantitative Real-time RT-PCR is a highly sensitive method, with demonstrable ability to detect mRNA expression differences from less than 0.01-fold to more than 100-fold. If a normal range can be established from non-diseased subjects, this apparent high degree of precision should prove useful for accurately discriminating patient values in the future in both early detection and treatment evaluation settings.

Selection of a housekeeping gene as an internal control is a key issue when RT-PCR is used to study mRNA expression [21]. In the present study, we selected *GAPDH *as an internal control. While mRNA expression of *GAPDH *may not be the same in different tissues [21], we believe that it was a satisfactory control gene for the present study because both tumor and matched normal esophageal tissues were used and the values for tumor and normal were both normalized to *GAPDH *as an initial step in the data analysis.

SPARC protein is a non-structural component of extracellular matrix-associated matricellular glycoprotein. It is known that SPARC protein is involved in the formation of focal adhesions and cytoskeletal structure, and that different levels influence cell adherence and migration capacity, potentially playing a role in the invasion and metastasis of tumor. *SPARC *has previously been shown to have increased mRNA expression in various human cancers, including ESCC, where it has been studied using array technology [22,23]. In the present study, we found increased mRNA expression of *SPARC *in 71% of patients. Yamahita et al also found high expression of *SPARC *mRNA in all 48 ESCC patients examined using Northern blot hybridization [24]. In another study, *SPARC *mRNA expression using the RT-PCR method was lowest in normal esophagus, intermediate in Barrett's esophagus, and highest in esophageal adenocarcinoma [25]. SPARC protein can be measured in human serum [26], thus, future study of the role of SPARC in ESCC should consider comparison of serum levels in cases and controls as an early step in assessing the potential value of SPARC protein as a biomarker for early detection or screening in a high-risk populations. Taken together, these results suggest that *SPARC *plays an important role in esophageal carcinogenesis and merits further investigation to determine how early in this process it becomes dysregulated.

*Fascin *is the first of the *fascins *to be cloned and is a highly conserved 55-KD actin-bundling protein. *Fascin *functions in the organization of two major forms of actin-based structures: dynamic cortical cell protrusions, and cytoplasmic microfilament bundles (eg, filopodia, spikes, lamellipodial ribs, dendrites and microvilli) [27]. The expression of *fascin *in epithelial neoplasms has been described only recently. In normal epithelial cells, *fascin *expression is usually absent or very low, but is often up-regulated in several types of human cancers, such as colon [28], breast [29], lung [30], and gastric [31]. To date, it has not been reported in ESCC. In a pilot study, we procured normal epithelia, dysplasia, and tumor from each of 11 esophagectomized ESCC patients and examined fascin protein expression by immunohistochemistry. Staining was strongest in tumor, intermediate in dysplasia, and absent in normal epithelia (unpublished data). These results suggest that the mRNA and protein expression patterns for *fascin *are similar, and that *fascin *could be a useful biomarker in ESCC patients.

CK4, a type II keratin, is typically paired with CK13 in expression in non-keratinized stratified epithelia of the upper digestive tract. Mutation of the *CK4 *gene can cause White Sponge Nevus, a benign autosomal dominant disorder that presents as leukokeratosis, usually in the mouth, but has also been reported in the esophagus and anogenital mucosa [32]. While CK4 protein alteration has been observed in many upper digestive tract tumors, including adenocarcinoma of the esophagus [33], it has not previously been reported in ESCC. In another pilot study, we found that CK4 protein expression, compared to normal epithelia, was decreased in 80% of dysplastic epithelia and 85% of invasive tumors (unpublished data). The reason *CK4 *expression is decreased in ESCC is unclear, but decreased expression of *CK4 *does influence the formation of cytoskeletal cells and this change may play a role in the development of tumor.

*EVPL*, *PPL*, and *TGM *are all involved in stabilizing the cornified cell envelope and, like *CK4 *discussed above, were all under-expressed in the ESCC patients evaluated in this study. Other investigators have shown that the C-terminus of *PPL *plays an important role in linking *PPL *and *EVPL *to intermediate filaments [34]. *EVPL *is a candidate gene for the tylosis esophageal cancer (TOC). Although our previous studies showed familial aggregation of ESCC in Shanxi Province, China [35,36], no cases with tylosis were identified in these high-risk families. In the present study, decreased *PPL *mRNA expression was significantly associated with a FH of UGI cancer. These results suggest that additional examination of the relationship between members of the *plakin *family of genes, particularly in familial ESCC, may be illuminating.

There are no immediate clinical applications from the findings reported here. Although our study size was only modest, the lack of association with tumor characteristics related to survival suggests that these nine genes are not likely to be useful in predicting outcome or suggesting specific therapies. Further evaluation of expression in relation to actual survival is needed to firm up this conclusion, however. For the moment, the greatest clinical potential for the nine genes studied here is their potential as early detection markers.

Although the nine genes studied in present study were well characterized at the mRNA level, changes at the protein level remain much less well characterized. Thus, logical next steps in future studies of these genes in ESCC are to further correlate protein expression status with mRNA expression, to evaluate the relation between protein expression and tumor phenotype (ie, grade, differentiation), to study the importance of cell location in protein over-expression, and to assess the relation of each of these factors to patient prognosis. In addition, we need to learn when in the ESCC carcinogenesis process dysregulation occurs if we are to determine the potential value of these genes as biomarkers for the early detection of ESCC.

## Conclusion

Results of the current study confirm that each of the nine genes evaluated are significantly dysregulated in the majority of ESCC cases and merit further investigation as potential susceptibility and early detection markers.

## Competing interests

The author(s) declare that they have no competing interests.

## Authors' contributions

NH conceived of study, oversaw laboratory and statistical analysis, and drafted the manuscript. LQ, J-ZS, and CW performed the laboratory analyses and conducted initial statistical analyses. CG contributed to the study design, aided in statistical analyses, and managed study data. Q-HW and YW oversaw the data and sample collection procedures for the study. AMG conceived of the study, participated in statistical analyses, and revised the manuscript. ME-B conceived of the study, aided in interpretation of the data, and revised the manuscript. PRT conceived of the study, oversaw laboratory and statistical analyses, revised the manuscript, and obtained funding. All authors read and approved the final manuscript.

## Pre-publication history

The pre-publication history for this paper can be accessed here:



## Figures and Tables

**Table 1:** Information on the nine genes examined: location, function, primers and probes

**No**	**Gene**	**LocusLink**	**Location**	**Putative function**	**Catalogue number for primer & probe (ABI)**
1	***SPARC***	6678	5q31.3-q32	Extracellular matrix remodeling	Hs00277762-m1
2	***FADD***	8772	11q13.3	Death receptor/cytoplasmic response	Hs00538709-m1
3	***Fascin***	6624	7p22	Cytoskeleton/regulation of cellular	Hs00979629-g1
4	***COL7A1***	1294	3p21.1	Extracellular matrix	Hs00164310-m1
5	***CK4***	3851	12q12-q13	Cytoskeleton	Hs00361611-m1
6	***TGM3***	7053	20q11.2	Protein modification	Hs00162752-m1
7	***ECM1***	1893	1q21	Extracellular matrix	Hs00189435-m1
8	***PPL***	5493	16p13.3	Cytoskeleton	Hs00160312-m1
9	***EVPL***	2125	17q25	Epidermal differentiation and maintenance	Hs00157430-m1
10	***GAPDH***				Hs99999905-m1

**Table 2:** Demographic and clinical characteristics of ESCC patients (N = 75)

**Characteristic**	**Males (N = 42)**	**Females (N = 33)**	**All (N = 75)**
**Age **(median)	57 yrs	55 yrs	56 yrs
**Tumor location**			
Lower	10 (24%)	4 (12%)	14 (19%)
Middle	30 (71%)	27 (82%)	57 (76%)
Upper	2 (5%)	2 (6%)	4 (5%)
**Tumor grade**			
I	4 (10%)	2 (6%)	6 (8%)
II	28 (67%)	23 (70%)	51 (68%)
III	10 (24%)	8 (24%)	18 (24%)
**Tumor stage**			
1	1 (2%)	0 (0%)	1 (1%)
2	7 (17%)	6 (18%)	13 17%)
3	33 (79%)	27 (82%)	60 (80%)
4	1 (2%)	0 (0%)	1 (1%)
**Metastases **(yes)	19 (45%)	15 (45%)	34 (45%)
Family history of UGI* cancer (yes)	12 (29%)	11 (33%)	23 (31%)
**Tobacco use **(yes)	34 (81%)	5 (15%)	39 (52%)
**Alcohol drinking **(yes)	10 (24%)	0 (0%)	10 (13%)

*Upper gastrointestinal

**Table 3:** mRNA expression of nine genes in ESCC patients

**Gene**	**Total number of cases**	**Distribution of N-fold mRNA expression**					**No. (%) over-expressed (N-fold ≥2.0)**	**No. (%) in "normal" expression range (N-fold range 0.5001–1.9999)**	**No. (%) under-expressed (N-fold ≤0.5)**	N-fold change from previous cDNA array study (N = 19)**
		**Min**	**25^th ^%**	**50^th ^% (median)**	**75^th ^%**	**Max**				

***SPARC***	75	0.1041	1.7695	4.4588	12.7286	91	53 (71%)	18 (24%)	4 (5%)	2.08
***FADD***	75	0.0055	1.5801	2.9897	6.4531	30	47 (63%)	25 (33%)	3 (4%)	2.04
***Fascin***	71*	0.0033	1.6896	5.1220	12.7878	1479	50 (70%)	10 (14%)	11 (15%)	2.22
***COL7A1***	75	0.0042	0.8746	3.0525	13.1775	285	43 (57%)	21 (28%)	11 (15%)	2.31

***CK4***	75	0.0000	0.0023	0.0206	0.1528	676	5 (7%)	8 (11%)	62 (83%)	0.16
***TGM3***	75	0.0000	0.0015	0.0209	0.3585	35858	6 (8%)	11 (15%)	58 (77%)	0.21
***ECM1***	75	0.0012	0.0159	0.0410	0.5236	140	7 (9%)	13 (17%)	55 (73%)	0.50
***PPL***	75	0.0076	0.0786	0.2306	0.8706	30	10 (13%)	15 (20%)	50 (67%)	0.41
***EVPL***	75	0.0040	0.0762	0.1856	0.9954	181	13 (17%)	12 (16%)	50 (67%)	0.48

*N = 71 as frozen tissue was exhausted

**Su et al, Cancer Res 2003;63:3872-6.

**Table 4:** mRNA expression of nine genes in ESCC patients by family history of uppergastrointestinal cancer

**Gene**	**Total number of cases**	**Median of N-fold mRNA expression**		
		**FH (+) cases (N = 23)**	**FH (-) cases (N = 52)**	*P*-value**

***SPARC***	75	5.3394	3.8266	0.099
***FADD***	75	4.2772	2.9452	0.125
***Fascin***	71*	6.7426	4.7068	0.515
***COL7A1***	75	3.6892	2.7104	0.904

***CK4***	75	0.0116	0.0378	0.143
***TGM3***	75	0.0028	0.0312	0.060
***ECM1***	75	0.0213	0.0938	0.038
***PPL***	75	0.1205	0.3353	0.044
***EVPL***	75	0.1247	0.3847	0.082

*N = 71 as frozen tissue was exhausted

**P-value from two-sided Wilcoxon rank sum test
